# Supplementary material for: The Impact of Mindfulness on Functional Brain Connectivity and Peripheral Inflammation in Breast Cancer Survivors with Cognitive Complaints
Source: Cancers (Basel). 2023 Jul 15;15(14):3632. doi: 10.3390/cancers15143632 (PMC10377401; doi:10.3390/cancers15143632)
Supplement: Supplementary file 1 [file cancers-15-03632-s001.zip › cancers-2409695-supplementary.pdf]

Supplementary Materials

The impact of mindfulness on functional brain connectivity and peripheral inflammation in breast cancer survivors with cognitive complaints

Questionnaires for Correlation Analysis

Our primary outcome measure was self-reported cognitive complaints as measured with the Cognitive Failure Questionnaire (CFQ) (Broadbent et al., 1982). The CFQ consists of 25 items assessing self-reported cognitive failures in daily activities, such as forgetting what the person was planning to do. Subscales on distraction, distraction in social situations, names and wordfinding, orientation, and a total summary score are available. Four extra questions assess whether symptoms increased over the past five years. The total score was used, with higher scores reflecting more cognitive complaints. We calculated Cronbach's alpha (and accompanying 95% confidence intervals) as a measure of internal consistency in R version 4.0.3 (ltm) (Rizopoulos, 2007). Cronbach's alpha ranges between 0 and 1, with higher values indicating higher reliability. The scale showed good internal consistency (α= 0.863; 95% CI=[0.846, 0.875]) in our sample. Additionally, depression, anxiety, and stress were measured with the Depression Anxiety Stress Scale (DASS) (Lovibond, 1995). We refer to the total score as a measure of emotional distress, with higher scores indicating more depression, anxiety, and stress. The DASS showed excellent internal consistency (α= 0.906; 95% CI=[0.878, 0.924]) in our sample. Furthermore, fatigue was evaluated with the Checklist Individual Strength (CIS) (Vercoulen et al., 1994). The total score was used, with higher scores reflecting more fatigue. The CIS showed excellent internal consistency (α= 0.911; 95% CI=[0.893, 0.923]) in our sample.

Supplementary Tables

Table S1. List of regions of interest within the included networks.

Network	Region of interest (MNI coordinates)	Size of ROI (number of voxels 2mm^3)
Default mode network	MPFC (1, 55, -3)	1346
	LP L (-39, -77, 33)	1041
	LP R (47, -67, 29)	1326
	PCC (1, -61, 38)	4833
Salience network	ACC (0, 22, 35)	1063
	AInsula L (-44, 13, 1)	446
	AInsula R (47, 14, 0)	388
	RPFC L (-32, 45, 27)	1166
	RPFC R (32, 46, 27)	581
	SMG L (-60, -39, 31)	233
	SMG R (62, -35, 32)	284
Dorsal Attention network	FEF L (-27, -9, 64)	88
	FEF R (30, -6, 64)	54
	IPS L (-39, -43, 52)	3285
	IPS R (39, -42, 54)	3137
Frontoparietal network	LPFC L (-43, 33, 28)	1703
	PPC L (-46, -58, 49)	832
	LPFC R (41, 38, 30)	1758
	PPC R (52, -52, 45)	837
Hippocampus	Left (59, 54, 27)	766
	Right (31, 57, 25)	703

MPFC: medial prefrontral cortex; LP: lateral parietal cortex; PCC: posterior cingulate cortex; ACC: anterior cingulate cortex; AInsula: anterior insula; RPFC: rostral prefrontal cortex; SMG:

supramarginal gyrus; FEF: frontal eye fields; IPS: intraparietal sulcus; LPFC: lateral prefrontal cortex; PPC: posterior parietal cortex; ROI: region of interest.

Table S2. Number of participants that received chemotherapy regimen per group.

**Chemotherapy regimen** *N* **(%)**	**Mindfulness (*n*=43)**	**Physical training (*n*=37)**	**Waitlist (*n*=38)**
Anthracyclines	1 (2.3)	1 (2.7)	0 (0.0)
Taxanes	10 (23.3)	9 (24.3)	8 (21.1)
Anthracyclines + Taxanes	32 (74.4)	27 (73.0)	30 (78.9)

Table S3. Results from multilevel mixed models showing significant intervention effects on functional network organization over time.

	**Estimate**	**SE**	***p***	**95% CI**
Characteristic path length				
Group-by-time interaction effects with waitlist as reference group				
Intercept	-0.38	0.20	.04*	[-0.80, -0.03]
t2 x Mindfulness	-0.25	0.27	.29	[-0.95, 0.22]
t3 x Mindfulness	-0.48	0.27	.08	[-1.04, 0.11]
t2 x Physical training	-0.92	0.31	.01*	[-1.56, -0.30]
t3 x Physical training	-1.20	0.32	.03*	[-1.87, -0.52]
Within group effects				
Intercept	0.22	0.17	.17	[-0.10, 0.55]
t2:Mindfulness	-0.04	0.21	.73	[-0.43, 0.35]
t3:Mindfulness	-0.16	0.19	.52	[-0.62, 0.18]
t2:Physical training	-0.70	0.21	.01*	[-1.15, -0.18]
t3:Physical training	-0.82	0.22	.01*	[-1.32, -0.28]
t2:Waitlist	0.24	0.21	.30	[-0.20, 0.78]
t3:Waitlist	0.33	0.22	.08	[-0.09, 0.83]
Clustering coefficient				
Group-by-time interaction effects with waitlist as reference group				
Intercept	0.02	0.20	.80	[-0.43, 0.35]
t2 x Mindfulness	0.01	0.29	.99	[-0.71, 0.51]
t3 x Mindfulness	-0.78	0.28	.03*	[-1.36, -0.17]
t2 x Physical training	0.10	0.32	.80	[-0.56, 0.75]
t3 x Physical training	-0.82	0.34	.04*	[-1.51, -0.10]
Within group effects				
Intercept	0.35	0.17	.04*	[0.02, 0.68]
t2:Mindfulness	-0.35	0.22	.12	[-0.75, 0.06]
t3:Mindfulness	-0.65	0.20	.01*	[-1.13, -0.30]
t2:Physical training	-0.24	0.21	.29	[-0.71, 0.30]
t3:Physical training	-0.62	0.23	.04*	[-1.14, -0.05]
t2:Waitlist	-0.33	0.22	.23	[-0.79, 0.24]
t3:Waitlist	0.14	0.23	.40	[-0.29, 0.66]
Global efficiency				
Group-by-time interaction effects with waitlist as reference group				
Intercept	0.49	0.20	<.001***	[0.06, 0.84]
t2 x Mindfulness	0.44	0.27	.14	[-0.25, 0.91]
t3 x Mindfulness	0.46	0.26	.14	[-0.09, 1.05]
t2 x Physical training	1.14	0.30	<.001***	[0.51, 1.76]
t3 x Physical training	1.05	0.32	<.001***	[0.39, 1.73]
Group-by-time interaction effects with mindfulness as reference group				
Intercept	-0.18	0.17	.35	[-0.51, 0.14]
t2 x Physical training	0.70	0.28	<.001***	[0.17, 1.35]
t3 x Physical training	0.64	0.27	<.001***	[0.13, 1.35]
Within group effects				
Intercept	-0.18	0.17	.35	[-0.51, 0.14]

t2:Mindfulness	0.00	0.21	1.00	[-0.39, 0.39]
t3:Mindfulness	0.06	0.19	.80	[-0.39, 0.40]
t2:Physical training	0.72	0.20	<.001***	[0.28, 1.23]
t3:Physical training	0.72	0.22	<.001***	[0.22, 1.26]
t2:Waitlist	-0.41	0.21	.11	[-0.85, 0.13]
t3:Waitlist	-0.38	0.22	.13	[-0.80, 0.11]

*p<.05; ***p<.001; CI = confidence interval; SE = standard error; t2 = post-intervention; t3 = three-month follow-up.

Table S4. Results from multilevel mixed models showing significant intervention effects on inflammatory profiles over time.

**Principal component 1**	**Estimate**	**SE**	***p***	**95% CI**
Group-by-time interaction effects with waitlist as reference group				
Intercept	0.16	0.17	.34	[-0.16, 0.49]
t2 x Mindfulness	0.17	0.25	0.49	[-0.31, 0.66]
t3 x Mindfulness	0.20	0.26	0.45	[-0.31, 0.70]
t2 x Physical training	0.58	0.26	0.03*	[0.07, 1.09]
t3 x Physical training	0.43	0.27	0.11	[-0.09, 0.94]
Within group effects				
Intercept	0.07	0.16	0.68	[-0.24, 0.38]
t2:Mindfulness	-0.18	0.17	0.31	[-0.51, 0.16]
t3:Mindfulness	-0.12	0.18	0.50	[-0.48, 0.23]
t2:Physical training	0.23	0.19	0.22	[-0.13, 0.60]
t3:Physical training	0.11	0.19	0.57	[-0.26, 0.48]
t2:Waitlist	-0.35	0.19	0.06	[-0.71, 0.01]
t3:Waitlist	-0.32	0.19	0.10	[-0.69, 0.05]

*p<.05; CI = confidence interval; SE = standard error; t2 = post-intervention; t3 = three-month follow-up.

Table S5. Spearman correlation between significant changes in inflammatory profiles/graph measures and changes on self-report questionnaires over time over all groups.

**Variable 1 (change scores)**	**Variable 2 (change scores)**	**Spearman correlation**	***p***
t2-t1			
PC 1	CFQ	0.08	.48
PC 1	DASS	0.13	.26
PC 1	CIS	-0.06	.61
PC 1	Characteristic path length	0.12	.32
PC 1	Global efficiency	-0.21	.08
Characteristic path length	CFQ	0.19	.11
Global efficiency	CFQ	-0.22	.06
Characteristic path length	DASS	0.12	.31
Global efficiency	DASS	-0.16	.17
Characteristic path length	CIS	0.22	.06
Global efficiency	CIS	-0.20	.09
t3-t1			
Characteristic path length	CFQ	0.13	.29
Clustering coefficient	CFQ	0.11	.35
Global efficiency	CFQ	-0.09	.47
Characteristic path length	DASS	0.18	.13
Clustering coefficient	DASS	0.13	.28
Global efficiency	DASS	-0.18	.13
Characteristic path length	CIS	0.18	.13
Clustering coefficient	CIS	0.20	.09
Global efficiency	CIS	-0.10	.40

CFQ = cognitive failure questionnaire; CIS = checklist individual strength; DASS = depression anxiety stress scale; PC 1 = principal component 1; t2-t1 = immediately post-intervention compared to baseline; t3-t1 = 3 months post-intervention compared to baseline.

Supplementary Figures

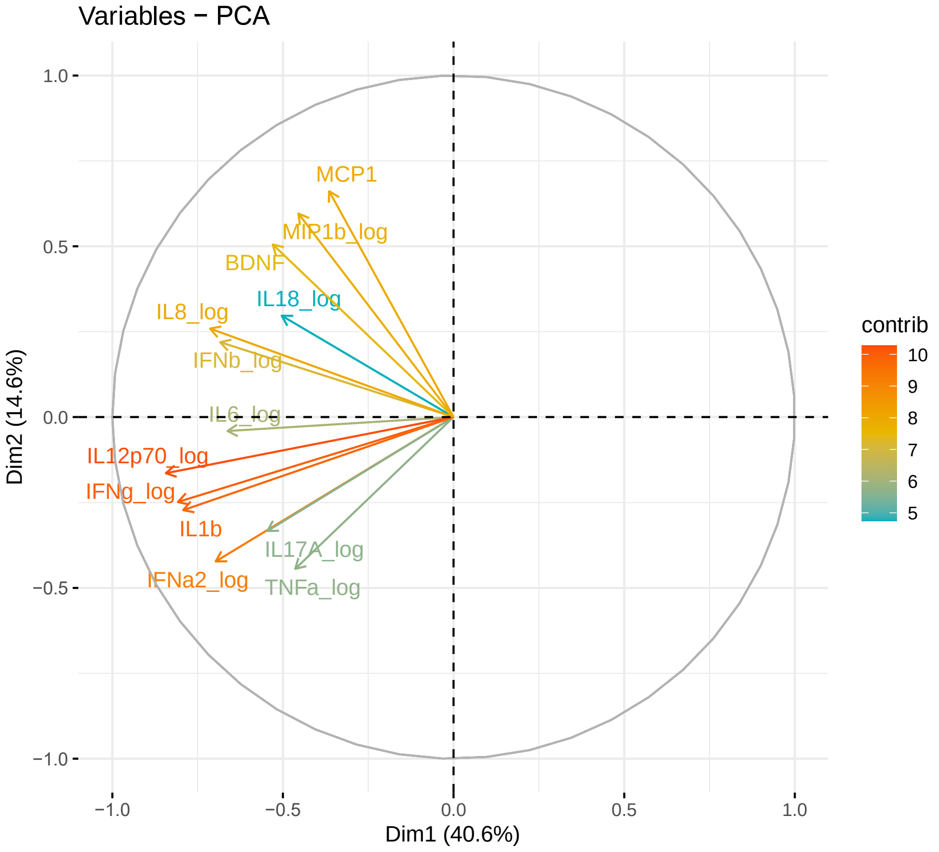

Figure S1. Graph of principal component 1 and 2, with vector arrows representing the coefficients of the variables on the principal components, colored by their contribution.

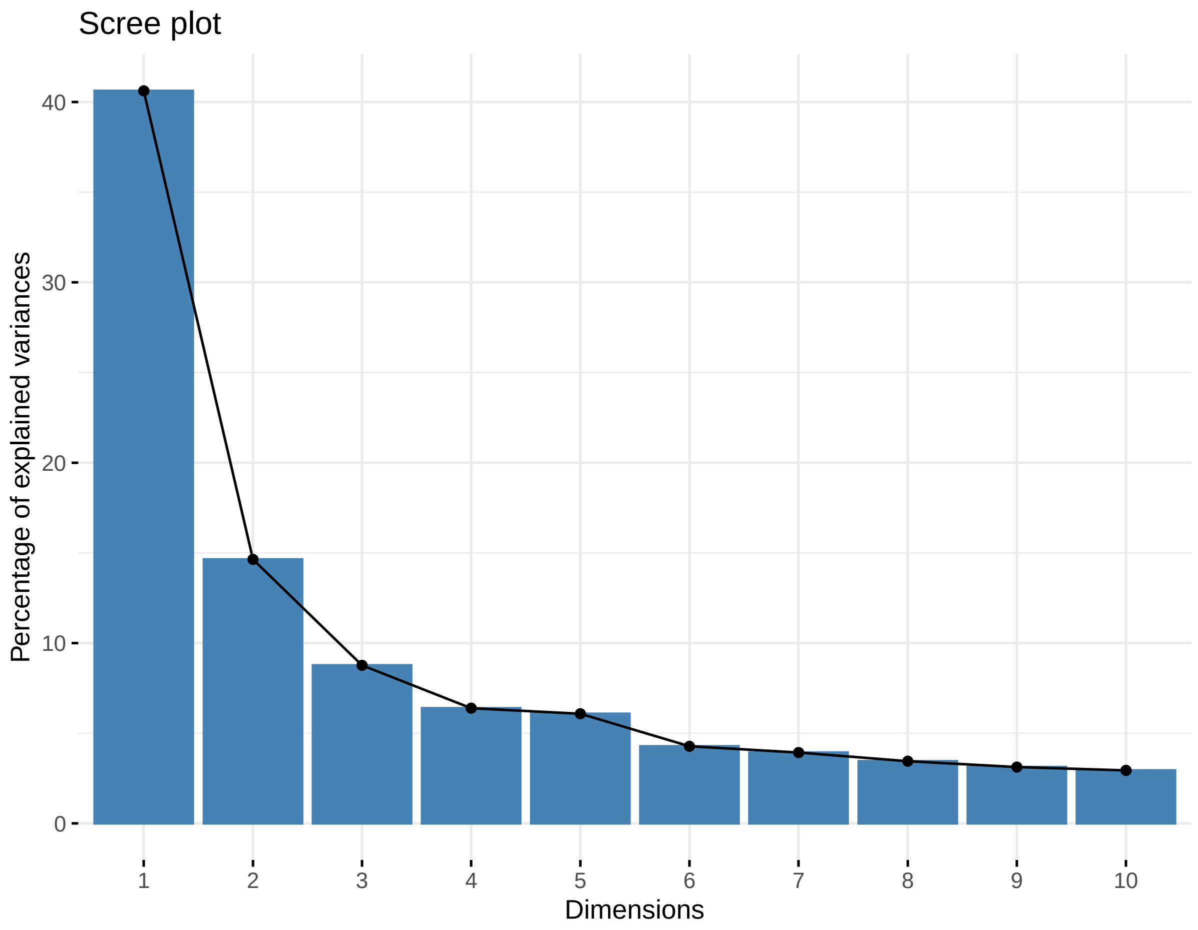

Figure S2. Scree plot showing the percentage of explained variances for each principal component.

References

1. Broadbent, D. E., Cooper, P. F., FitzGerald, P., & Parkes, K. R. (1982). The Cognitive Failures Questionnaire (CFQ) and its correlates. *The British Journal of Clinical Psychology*, *21*(1), 1–16. https://doi.org/10.1111/J.2044-8260.1982.TB01421.X
2. Lovibond, S. (1995). *Manual for the depression anxiety stress scales* (2nd ed.). Psychology Foundation.
3. Rizopoulos, D. (2007). ltm: An R package for latent variable modeling and item response analysis. *Journal of Statistical Software*, *17*(5), 1–25. https://doi.org/10.18637/JSS.V017.I05
4. Vercoulen, J. H. M. M., Swanink, C. M. A., Fennis, J. F. M., Galama, J. M. D., van der Meer, J. W. M., & Bleijenberg, G. (1994). Dimensional assessment of chronic fatigue syndrome. *Journal of Psychosomatic Research*, *38*(5), 383–392. https://doi.org/10.1016/0022-3999(94)90099-X
